# Sensory nerves directly promote osteoclastogenesis by secreting peptidyl-prolyl cis-trans isomerase D (Cyp40)

**DOI:** 10.1038/s41413-023-00300-w

**Published:** 2023-12-14

**Authors:** Junqin Li, Bin Liu, Hao Wu, Shuaishuai Zhang, Zhuowen Liang, Shuo Guo, Huijie Jiang, Yue Song, Xing Lei, Yi Gao, Pengzhen Cheng, Donglin Li, Jimeng Wang, Yang Liu, Di Wang, Nazhi Zhan, Jing Xu, Lin Wang, Guozhi Xiao, Liu Yang, GuoXian Pei

**Affiliations:** 1 Southern University of Science and Technology Hospital, No. 6019 Liuxian Street, Xili Avenue, Nanshan District, Shenzhen, 518055 China; 2 Department of Orthopaedics, Xijing Hospital, Air Force Medical University, Xi’an, 710032 China; 3 Department of Orthopedics, General Hospital of Northern Theater Command, No. 83, Wenhua Road, Shenhe District, Shenyang, 110016 China; 4 Department of Orthopaedics, Tangdu Hospital, Fourth Military Medical University, 710038 Xi’an, PR China; 5 Department of Biomedical Engineering, Fourth Military Medical University, 710032 Xi’an, PR China; 6 Lingtong Rehabilitation and Recuperation Center, Xi’an, 710600 China; 7 Department of Orthopedics, The Fourth Medical Center, Chinese PLA General Hospital, 100048 Beijing, PR China; 8 Department of Orthopedics, Linyi People’s Hospital, LinYi, 276000 China; 9 Department of Orthopedics, 81 Army Hospital of the People’s Liberation Army, Zhangjiakou, 075000 China; 10 Department of Anaesthesiology and Perioperative Medicine, Xijing Hospital, Fourth Military Medical University, Xi’an, 710032 China; 11 School of Medicine, Southern University of Science and Technology, Shenzhen, 518055 China

**Keywords:** Bone, Pathogenesis

## Abstract

Given afferent functions, sensory nerves have recently been found to exert efferent effects and directly alter organ physiology. Additionally, several studies have highlighted the indirect but crucial role of sensory nerves in the regulation of the physiological function of osteoclasts. Nonetheless, evidence regarding the direct sensory nerve efferent influence on osteoclasts is lacking. In the current study, we found that high levels of efferent signals were transported directly from the sensory nerves into osteoclasts. Furthermore, sensory hypersensitivity significantly increased osteoclastic bone resorption, and sensory neurons (SNs) directly promoted osteoclastogenesis in an in vitro coculture system. Moreover, we screened a novel neuropeptide, Cyp40, using an isobaric tag for relative and absolute quantitation (iTRAQ). We observed that Cyp40 is the efferent signal from sensory nerves, and it plays a critical role in osteoclastogenesis via the aryl hydrocarbon receptor (AhR)-Ras/Raf-p-Erk-NFATc1 pathway. These findings revealed a novel mechanism regarding the influence of sensory nerves on bone regulation, i.e., a direct promoting effect on osteoclastogenesis by the secretion of Cyp40. Therefore, inhibiting Cyp40 could serve as a strategy to improve bone quality in osteoporosis and promote bone repair after bone injury.

## Introduction

Sensory nerves detect damaging stimuli^1^ in tissues that are in close contact with the external environment, such as the skin, lung and gut; furthermore, they can regulate the subsequent immune response by releasing neuropeptides.^2–4^ Recent studies have further emphasized the important role of sensory nerves in regeneration and maintenance of homeostasis in the musculoskeletal system, which resides deeper within the body.^5–7^

Approximately 77% of neurochannels in bones are sensory endings^1^ that closely interact with osteoblasts^8^ and osteoclasts.^9^ Multiple studies have demonstrated the effect of sensory nerves on the formation of osteoblasts and bone.^10–12^ Recently, a direct mechanism was discovered by which sensory nerves regulate the mobilization of hematopoietic stem cells (HSCs) in the bone marrow.^13,14^ In patients with hereditary sensory neuropathy, the absence of osteoclasts in regions with severely degenerated sensory nerves highlights the critical role of sensory nerves in osteoclastogenesis.^15^ The impact of sensory neuropeptides such as calcitonin gene-related peptide (CGRP)^16,17^ and substance P (SP)^18,19^ on osteoclasts and bone resorption has been studied. However, presently, there is a lack of direct evidence in support of the influence of efferent sensory nerves on osteoclasts.

To address this gap in knowledge, we induced sensory hypersensitivity using resiniferatoxin (RTX) and applied viral vectors to trace the signals between the sensory nerves and osteoclasts. Furthermore, we conducted in vitro experiments to study efferent functions and mechanisms underlying the effect of sensory nerves on osteoclasts.

## Results

### Abundant efferent signals in sensory nerves are transported into bone cells

**Fig. 1:**
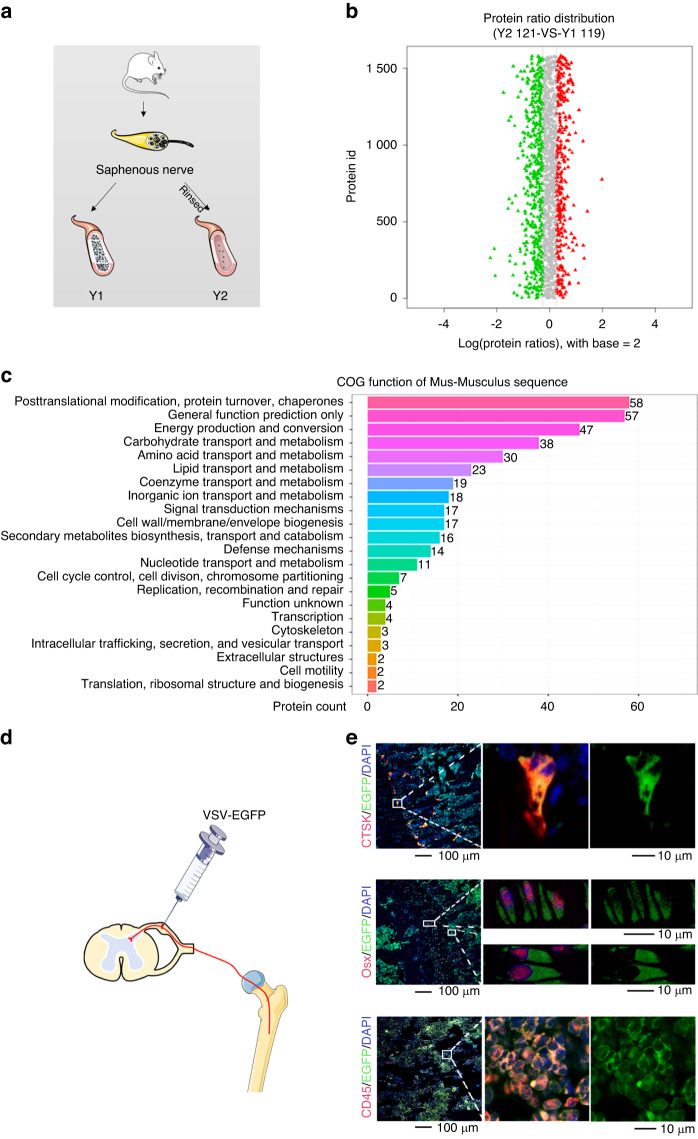
Abundant efferent signals in sensory nerves are transported into bone cells. **a**–**c** Screening of secreted proteins in sensory nerves. **a** Graphic illustration of grouping for iTRAQ. **b** Differentially expressed proteins between Y1 and Y2. The red dots indicate proteins that were more abundant in the Y2 group (141 proteins), while the green dots represent proteins that are more abundant in the Y1 group (237 proteins). **c** COG functional classification of proteins that were more abundant in Y2, which were considered to be axoplasmic proteins. **d**, **e** Anterograde tracing of signals from the sensory nerve to the bone. **d** The schematic illustrates the process of injecting VSV-EGFP into the DRG (dorsal root ganglia) at L3 and L4. After 5 days, the femur was harvested for immunofluorescence staining (**e**)

Nerve functions are modulated by the release of neuropeptides^20,21^; therefore, in the present study, our focus was on functional neuropeptides of sensory nerves and their impact on bone. Since functional neuropeptides synthesized in the dorsal root ganglion (DRG) are transported peripherally and stored in axons before being released to target cells,^22,23^ they are considered abundant components in the axoplasm. We employed the iTRAQ technique for proteins present in the saphenous nerve axoplasm (Fig. 1a). The iTRAQ technique uses a variety of isotope reagents for labeling the N-terminal or lysine side chain groups of protein polypeptides. Thereafter, the labeled peptides were analyzed using a high-precision mass spectrometer, which allows for identification of multiple proteins and provides reliable quantitative proteome information.^24^

In total, 237 proteins were identified in the saphenous nerve axoplasm (Fig. 1b, Supplement Excel 1, and Supplement Excel 2). The functional classification of these axoplasmic proteins was performed using Clusters of Orthologous Groups (COG) (Fig. 1c). Among the identified proteins, 58 were involved in post-translational modifications and protein turnover or acted as chaperones, which suggested their crucial roles in the maintenance of normal cell functions. Additionally, 57 proteins were predicted to have general functions, thereby indicating their involvement in various cellular processes, such as intracellular signaling, basic metabolism, and maintenance of cell structure. Furthermore, 47 proteins were associated with energy production and conversion and potentially participated in cellular respiration, ATP synthesis, and other energy metabolism pathways. Moreover, the presence of 38 proteins associated with carbohydrate transport and metabolism suggested their participation in the uptake, transport, and metabolism of glucose and other carbohydrates for the maintenance of energy supply. Finally, 30 proteins, which were associated with amino acid transport and metabolism, may play key roles in amino acid transport, synthesis, and degradation, thereby supporting normal cellular functions and protein synthesis.

The DRG is composed of afferent sensory fiber cells.^25^ Anterograde transport from the sensory nerves to the bone can be effectively tracked by administering VSV-EGFP into the DRG (Fig. 1d and Fig. S1a). VSV was designed to specifically infect neurons as well as for anterograde transsynaptic tracing^26^ (Fig. S1b). Five days after infection, we collected femur samples to perform immunofluorescence staining and found abundant signals in osteoclasts (cathepsin K, CTSK^+^), osteoblastic cells (osterix, Osx^+^), and leukocytes (cluster of differentiation 45, CD45^+^) in the bone (Fig. 1e). We also traced sensory signals in the muscle tissue that tightly enveloped the bone. After entering the muscles, the majority of the sensory signals (87.28%) were found in fast-twitch myofibers, which exhibited rapid bursts of contraction and fatigued quickly. Conversely, only a small portion of the signals (12.72%) were distributed in slow-twitch myofibers, which exerted slow contractions (Fig. S1c).^27^ The distinctive uneven and dominant distribution in the fast-twitch fibers suggested that the efferent function of sensory nerves may primarily involve rapid adjustment processes.

In conclusion, the presence of abundant secretory signals from the sensory nerves that penetrate into the bone suggests a functional impact of these nerves on the bone.

### Sensory hypersensitivity induces osteopenia in mice

**Fig. 2:**
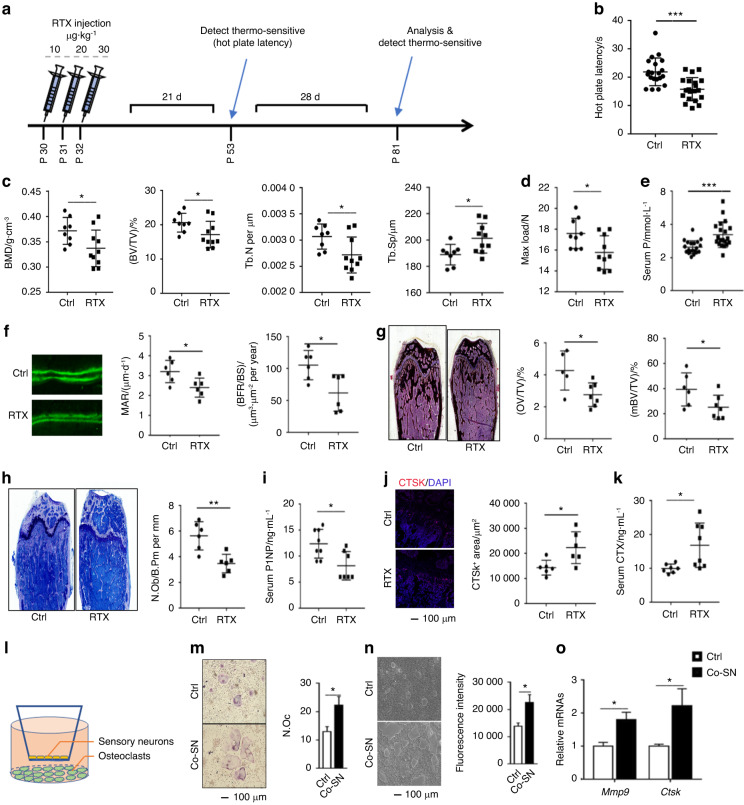
Sensory hypersensitivity induces osteopenia in mice. **a** Graphic illustration of RTX injection to induce sensory hypersensitivity. **b** Sensory activation was assessed by hot plate latency. **c** Quantitative analyses of BMD, BV/TV, Tb.Sp, and Tb.N of the tibia by μCT. **d** Maximal loading of femur by three-point bending assays. **e** Serum inorganic phosphorus concentration. **f** Representative images of calcein double-labeling of the trabecular bone of the femur, with quantification of the mineral apposition rate (MAR) and bone formation rate per bone surface (BFR/BS). **g** Von Kossa staining and quantitative analysis of osteoid volume per tissue volume (OV/TV) and mature bone volume per tissue volume (mTV/BV) in the femoral bone tissue. **h** Toluidine blue staining and quantitative analysis of the number of osteoblasts per trabecular bone perimeter (N.Ob/B.Pm). **i** ELISA analysis of the serum P1NP levels. **j** Representative confocal images and quantitative analysis of CTSK in bone. **k** ELISA analysis of the serum CTX levels. **l**–**o** Isolated coculture of SN and osteoclasts. **l** Graphic illustration of the isolated coculture system. **m** Representative TRAP staining pictures. TRAP^+^ osteoclasts with more than three nuclei were quantified using ImageJ software. **n** Resorptive activity was measured by plating cells on fluorescent calcium phosphate-coated plates. **o** Expression of the resorption-related genes *Mmp9* and *CTSK* in osteoclasts. *P < 0.05, **P < 0.01, ***P < 0.001, versus controls, Student’s *t* test. The results are expressed as the mean ± s.d

We induced sensory hypersensitivity in the mice by injecting them with RTX to investigate the potential efferent influences of sensory nerves on bone homeostasis (Fig. 2a, b). Microcomputed tomography (μCT) analysis of the tibia revealed that key parameters, such as bone mineral density (BMD), bone volume/tissue volume fraction (BV/TV), trabecular number (Tb.N), and trabecular separation (Tb.Sp), were significantly altered in the mice with sensory hypersensitivity compared to their control littermates (Fig. 2c). However, trabecular thickness (Tb.Th), trabecular bone surface/bone volume (Tb.BS/BV), and all the key parameters in the cortical bone were not significantly altered (Fig. S2a). The three-point bending test also showed decreased bone quality (Fig. 2d). Furthermore, the serum biochemical analyses showed a dramatic reduction in serum inorganic phosphorus (P, Fig. 2e). Since abnormalities in the levels of serum P are typically associated with renal dysfunction, glucose metabolism, and lipid metabolism, we conducted tests on serum biochemical markers (Fig. S2b, c) that are related to metabolism and renal function. However, none of these markers showed any significant alteration in the RTX-treated mice. These findings strongly suggest that the sensory nerves exert efferent effects on bone homeostasis.

A reduction in bone formation and the mineral apposition rate in the RTX-treated mice was confirmed by calcein double-labeling (Fig. 2f). Von Kossa staining showed decreased osteoid in the RTX-treated mice (Fig. 2g). Furthermore, toluidine blue staining showed a decrease in osteoblast function and bone formation in the RTX-treated mice (Fig. 2h). Accordingly, the level of procollagen type N-terminal propeptide (P1NP, Fig. 2i) in the serum was significantly decreased. All these findings were consistent with our results of in vitro experimentation published previously, which showed that sensory nerves exerted direct efferent regulation on the differentiation of bone marrow stem cells (BMSCs).^9^

Thereafter, we focused on bone resorption and found that the number of CTSK^+^ osteoclasts was significantly elevated in the RTX-treated mice (Fig. 2j). Accordingly, the level of the osteoclast bone resorption marker carboxy-terminal collagen crosslinks (CTXs) was increased significantly in the serum (Fig. 2k). To further elucidate the mechanism of action of sensory nerves, we purified sensory neurons (SNs) from the DRG (Fig. S3a). The SNs were isolated and cocultured with osteoclasts such that any direct cell contact was avoided (Fig. 2l). Notably, the presence of SNs in the culture significantly increased the number of osteoclasts (Fig. 2m), osteoclast resorption activity (Fig. 2n), and the expression of resorption-related genes (Fig. 2o). These results suggested that sensory nerves can directly promote osteoclastogenesis. However, there was no change in the number of PGP9.5^+^ peripheral neurons (Fig. S2d) in the bones of the RTX-treated mice, which further underscored the importance of functional neuropeptides of sensory nerves for bone.

### Cyp40 is crucial for the promotion of osteoclastogenesis by sensory nerves

**Fig. 3:**
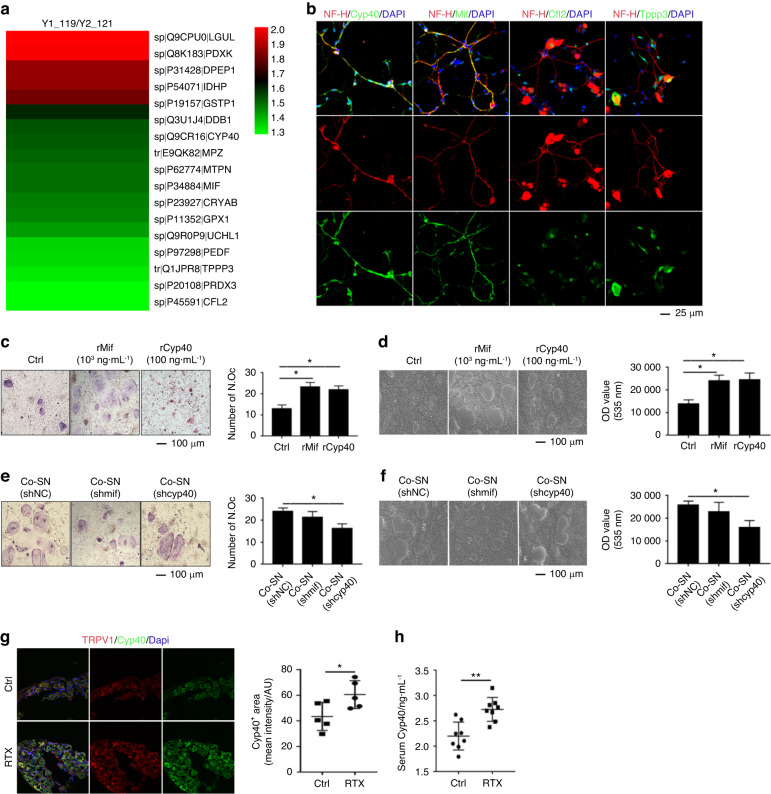
Cyp40 is crucial in sensory nerves that elevate osteoclastogenesis. **a** Saphenous nerve axoplasm proteins related to proliferation and differentiation were identified. These proteins include Cfl2, Tppp3, Aacs, Pedf, Glrx3, Prdx2, Uchl1, Gpx1, Adt2, Mtpn, Mif, Cryab, Cyp40, Mk03, Pgk1, and Ddb1. **b** Immunofluorescence staining of identified saphenous nerve axoplasm proteins (Cyp40, Mif, Cfl2, and Tppp3). **c**, **e** TRAP staining of osteoclasts and TRAP-positive multiple nucleated cells with ≥3 nuclei per well were scored (*n* = 3). **d**, **f** Resorptive activity was measured by plating BMMCs on fluorescent calcium phosphate-coated plates. **g** Representative confocal images and quantitative analysis of the DRG from mice injected with RTX or vehicle solution (Ctrl). **h** ELISA analysis of serum Cyp40 levels. These experiments were repeated in 3 independent biological replicates, each with 3 technical replicates. *P < 0.05, **P < 0.01, ***P < 0.001, and N.S. means not significant, versus controls, Student’s *t* test. The results are expressed as the mean ± s.d

Since sensory nerves play a direct role in the promotion of osteoclastic differentiation (Fig. 2l–o), we focused on the neuropeptides in the sensory axoplasm related to the proliferation and differentiation of osteoclasts. We investigated and identified 17 axoplasmic components that were related to these processes (Fig. 3a). Among these, the components selected for validation included Cyp40, macrophage inhibitory factor (Mif), cofilin 2 (Cfl2), and tubulin polymerization promoting protein family member 3 (Tppp3); moreover, Cyp40 and Mif were detected in the axons (Fig. 3b). Notably, osteoclasts and multiple immune cells are differentiated from HSCs, and a comprehensive investigation of the relevant literature revealed a close association between Cyp40 and Mif and immune cells, as reported in previous studies.^28–31^ Therefore, Mif and Cyp40 were selected for subsequent studies.

Both Cyp40 and Mif increased osteoclastic differentiation (Fig. 3c) and the resorptive activity of osteoclasts (Fig. 3d). To further study their roles in the promotion of osteoclastogenesis by sensory nerves, we downregulated Cyp40 (shCyp40) and Mif (shmif) in the SNs (Fig. S3c). Thereafter, the osteoclasts were cultured with the modified SN. We observed that the downregulation of Cyp40 significantly attenuated the ability of SNs to promote osteoclastogenesis (Fig. 3e, f). However, the downregulation of Mif did not significantly affect the ability of the SN to promote osteoclastogenesis (Fig. 3e, f). Thus, we concluded that Cyp40 is crucial for the ability of the SN to promote osteoclastogenesis in vitro. The basal expression of Cyp40 in the sensory neurons was significantly higher than that in the bone cells (Fig. S3d). In addition, recombinant Cyp40 dramatically reduced osteoblastic differentiation (Fig. S3e), and these results confirmed the crucial role of Cyp40 in sensory nerve efferent functions.

In vivo, Cyp40 was found to be upregulated in the RTX-treated mice (Fig. 3g), which included an increase in the serum levels of Cyp40 (Fig. 3h). The combination of the aforementioned results clarified that Cyp40 plays a vital role in the efferent function of sensory nerves on the bone, specifically in the regulation of osteoclastogenesis.

### Cyp40 secreted by sensory neurons enters osteoclasts, inhibits the Ras/c-Raf/p-Erk signaling pathway and promotes osteoclastogenesis

**Fig. 4:**
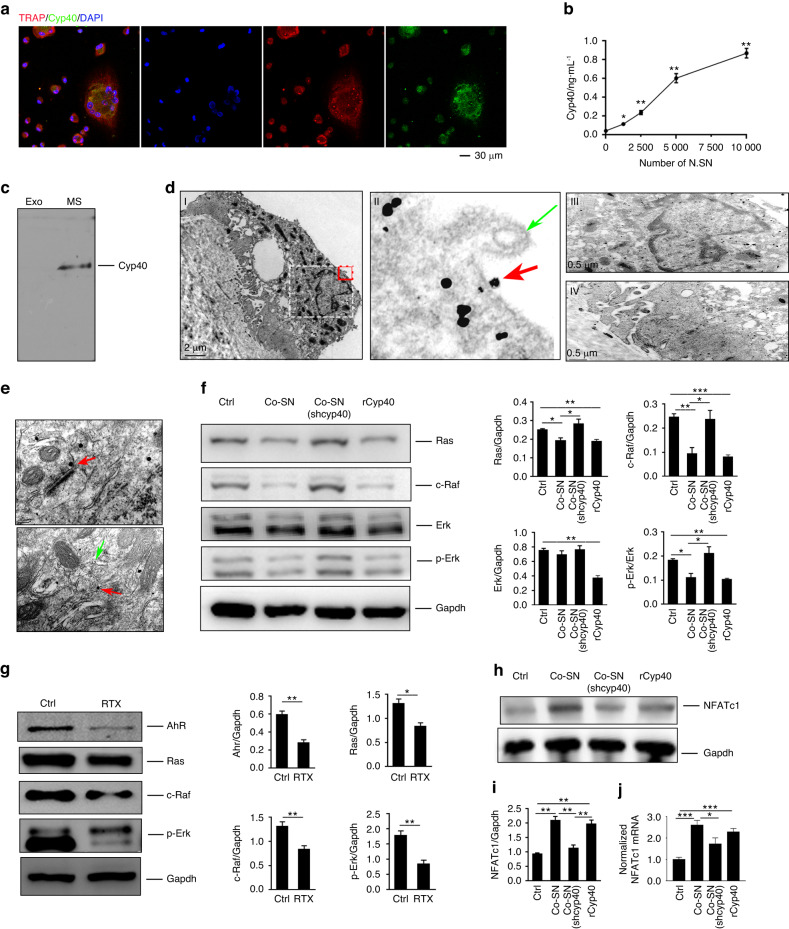
Cyp40 is a secreted factor that enters osteoclasts and inhibits Ras/c-Raf/p-Erk signaling, thereby promoting osteoclastogenesis. **a** Confocal images of osteoclasts isolated cocultured with SN that were transfected with plasmids containing Cyp40 tagged with EGFP. **b** ELISAs were performed to analyze the levels of secreted Cyp40 in the cell culture medium from varying numbers of sensory neurons (*n* = 3). **c**–**e** Cyp40 is secreted from sensory nerves and enters osteoclasts via a nonexosomal mechanism. To test this hypothesis, we separated the coculture medium into exosomal (Exo) and nonexosomal supernatants (MS), which were analyzed separately via Western blotting for the presence of Cyp40 (**c**). **d** Representative IEM images of the distribution of EGFP-tagged Cyp40 (black granules) in osteoclasts. Osteoclasts on the bone surface were cocultured with SN cells transfected with plasmids containing EGFP-tagged Cyp40. **e** Representative IEM images of EGFP-tagged Cyp40 (black granules, red arrow) in the brain and membrane vehicles (green arrow). **f** Western blotting of Ras, c-Raf, Erk, and p-Erk in osteoclasts, which were isolated and cocultured without (Ctrl), or with recombinant Cyp40 (rCyp40), SN (Co-SN), or Cyp40 knockout SN (Co-SN (shCyp40)). **g** Following the induction of osteoclast differentiation of BMMCs from RTX-treated or Ctrl mice, Western blot analysis was performed to determine the levels of AhR, Ras, c-Raf, and p-Erk proteins in the generated osteoclasts. **h**, **i** Western blotting of NFATc1 in osteoclasts, which were isolated and cocultured without (Ctrl), or with recombinant Cyp40 (rCyp40), SN (Co-SN), or Cyp40 knockout SN (Co-SN (shCyp40)). **j** qPCR analysis of NFATc1 mRNA (*n* = 3). *P < 0.05, **P < 0.01, ***P < 0.001, and N.S. means not significant, versus controls, Student’s *t* test. The results are expressed as the mean ± s.d

To confirm the release of Cyp40 from the SNs into osteoclasts, we constructed and transfected an EGFP-tagged Cyp40 vector (Fig. S4a) into the SNs. With the cocultivation of modified SNs with osteoclasts, we detected the presence of EGFP-tagged Cyp40 in osteoclasts (Fig. 4a), which confirmed that Cyp40 was indeed secreted by SN and taken up by osteoclasts. Furthermore, the ELISA results demonstrated a dose‒response curve of secreted Cyp40 in relation to the increasing numbers of SNs (Fig. 4b). These findings collectively provide substantial evidence that Cyp40 is secreted from the SNs.

The mechanism of transportation of Cyp40 between cells has not been studied previously^32^; therefore, we investigated the transfer of Cyp40 between cells. The coculture medium was analyzed, which revealed an absence of Cyp40 in the exosomes (Fig. 4c), indicating that Cyp40 did not travel between sensory neurons and osteoclasts via the exosomes. The results from IEM revealed that SN Cyp40 was present in the cell membrane (Fig. 4d I, II; red arrow) but was instead wrapped in vesicles (Fig. 4d I, II; green arrow). Upon entering osteoclasts, Cyp40 is ubiquitously distributed within cells. Furthermore, it was present at lower levels in the nucleus (Fig. 4d, III) and at higher levels throughout the cytoplasm (Fig. 4d I), including at the ruffled border, which is responsible for bone resorption (Fig. 4d IV). Due to the abundance of Cyp40 in neurons,^33^ we also examined its transport within the brain. The results revealed that during intercellular transport, Cyp40 was independently present in cell membranes (Fig. 4e, red arrow) rather than being enclosed by a membrane for transport support (Fig. 4e, green arrow). Thus, SN Cyp40 entered osteoclasts via a nonexosomal mechanism.

Furthermore, Ras/c-Raf/p-Erk signaling reportedly plays an important role in osteoclast survival, proliferation, apoptosis, formation, polarity, and differentiation.^34^ Therefore, we further examined whether the Ras signaling pathway was involved in the mechanism of regulation of osteoclasts by SN. Our findings revealed that SN could downregulate the expression of Ras and c-Raf as well as the phosphorylation of ERK in osteoclasts (Fig. 4f). Moreover, Cyp40 could downregulate Ras/c-Raf/p-Erk (Fig. 4f) expression. Notably, the absence of Cyp40 in the sensory neurons abolished their ability to downregulate Ras/c-Raf/p-Erk within the osteoclasts (Fig. 4f). In the RTX-treated mice, inhibition of Ras/c-Raf/p-Erk signaling was also observed in osteoclasts in vivo (Fig. 4g), which is opposite to the change observed in Cyp40 expression (Fig. 3g). The combination of the in vivo and in vitro results indicated that Cyp40 plays a crucial role in enabling sensory nerves to negatively regulate Ras/c-Raf/p-Erk in osteoclasts.

Reportedly, p-Erk inhibits osteoclastogenesis by downregulating the expression of NFATc1, which is a key transcription regulator in osteoclasts.^35–37^ Our findings demonstrated that NFATc1 upregulation and the subsequent increase in osteoclastogenesis occurred when p-Erk was downregulated in osteoclasts (Fig. 4f, h–j). Similarly, these alterations in the expression of NFATc1 were dependent on Cyp40 (Fig. 4h–j).

Taken together, the current findings indicate that sensory nerves promote osteoclastogenesis by secreting Cyp40, which results in the downregulation of Ras/c-Raf/p-Erk signaling and subsequently lifts the inhibition of NFATc1 by p-Erk and promotes osteoclastogenesis.

### Sensory nerves and Cyp40 promote osteoclastogenesis via AhR

**Fig. 5:**
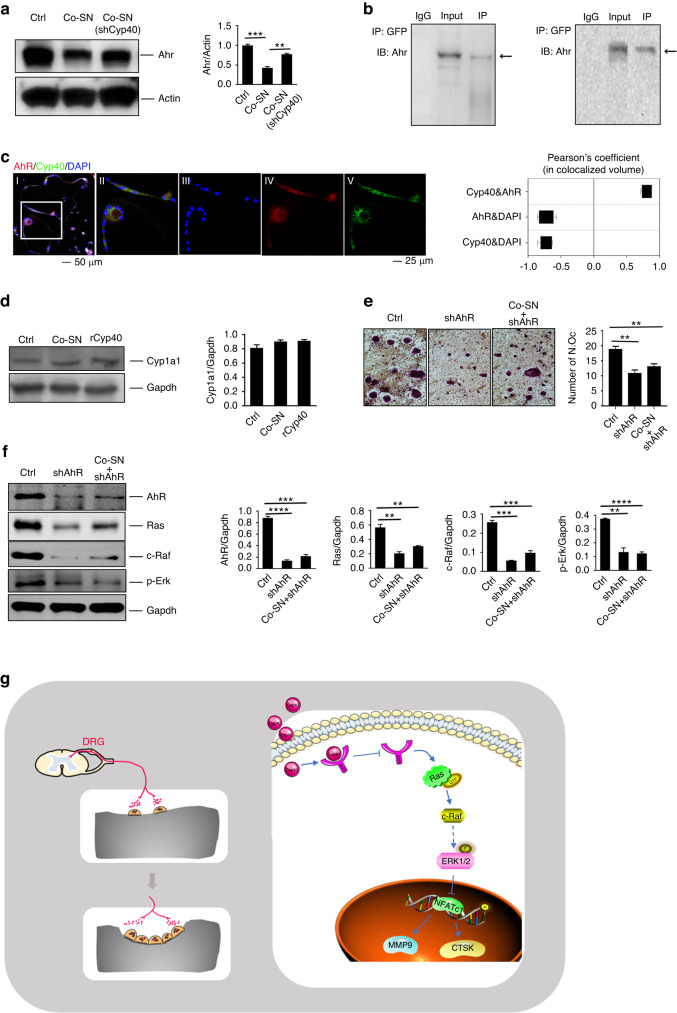
Cyp40 promotes osteoclastogenesis by downregulating AhR and activating AhR noncanonical activity. **a** Western blotting of AhR expression in osteoclasts. **b** Coimmunoprecipitation of EGFP-tagged Cyp40 from the SNs interacts with AhR in osteoclasts. **c** Representative confocal images and analysis of sensory Cyp40-GFP and AhR localization in osteoclasts. **d** Western blotting of Cyp1a1 in osteoclasts and osteoclasts cultured with SNs or recombinant Cyp40. **e** TRAP staining of osteoclasts and TRAP-positive multiple nucleated cells with ≥3 nuclei per well were scored (*n* = 3). **f** Western blotting of AhR, Ras, c-Raf, and p-Erk in osteoclasts, AhR knockdown osteoclasts (shAhR), or modified osteoclasts (shAhR) that had been cocultured with SN. **g** Summative figure illustrating the role of Cyp40 in bone. *P < 0.05, **P < 0.01, ***P < 0.001, and N.S. means not significant, versus controls, Student’s *t* test. The results are expressed as the mean ± s.d

Reportedly, AhR activates Ras, which further activates ERK to regulate cell proliferation and differentiation.^38^ Additionally, Cyp40 modulates AhR expression and distribution.^39^ Therefore, we investigated whether AhR was involved in the ability of sensory nerves to promote osteoclastogenesis. Initially, we found a significant decrease in the AhR levels in the RTX-treated mice (Fig. 4g). Furthermore, the AhR level in osteoclasts was downregulated by sensory neurons, and this downregulation was found to be Cyp40-dependent (Fig. 5a). Furthermore, an interaction between AhR and sensory neuronal Cyp40 was observed in osteoclasts (Fig. 5b). Immunofluorescence staining of osteoclasts revealed colocalization of AhR with neuron-derived Cyp40, predominantly in the cytoplasm (Fig. 5c). Furthermore, AhR exerts both canonical transcriptional activity in the nucleus and transcription-independent protein activity in the cytoplasm.^40^ Cytochrome P450 1A1 (Cyp1a1) serves as a hallmark for the canonical transcriptional activity of AhR (Fig. 5d). Notably, the promotion of osteoclastogenesis by SN and Cyp40 did not lead to any changes in Cyp1a1 expression. These findings suggest that AhR downregulation may be involved in the ability of sensory nerves to promote osteoclastogenesis through its interaction with Cyp40. Notably, this interaction involves the noncanonical rather than canonical activity of AhR.

Interestingly, direct AhR downregulation did not increase osteoclastogenesis, although it eliminated the promoting effect of sensory nerves on osteoclastogenesis; furthermore, the suppression of AhR expression resulted in a significant decrease in osteoclastogenesis (Fig. 5e). Notably, Ras/c-Raf/p-Erk signaling was also significantly downregulated with AhR inhibition (Fig. 5f). These findings implied that the maintenance of a basic level of AhR is crucial for normal osteoclastogenesis. When AhR is not sufficient, upstream molecules such as Cyp40 in sensory neurons are unable to identify functional targets, and consequently, they lose their regulatory role in osteoclasts. Collectively, the sensory Cyp40 binds to and downregulates AhR expression in osteoclasts while modulating its noncanonical activity, thereby ultimately promoting osteoclastogenesis.

In summary, sensory nerves promote osteoclastogenesis by secreting Cyp40. Furthermore, sensory Cyp40 binds to and downregulates the expression of AhR in osteoclasts while modulating its noncanonical activity. This results in a subsequent downregulation of the Ras/c-Raf/p-Erk signaling pathway, which relieves the inhibitory effect of p-Erk on NFATc1 and ultimately leads to an increase in osteoclastogenesis (Fig. 5g).

## Discussion

To avoid damage by environmental factors, sensory endings enable the rapid detection of environmental insults such as cold, heat or pain^41,42^; furthermore, sensory nerves are integral for the generation of immune responses to protect the body intact.^1^ Given their afferent functions, sensory neurons have been found to exert efferent influence and directly alter organ physiology.^43^ Gao X et al. found that sensory nerves directly regulated the maintenance of HSCs and the egress of the bone marrow.^1^ Similar functions of sensory nerves have been reported for dendritic cells in skin infections^3^ and for CD4^+^ and resident innate lymphoid type 2 cells in airway inflammation.^4^ Notably, T lymphocytes, dendritic cells, and osteoclasts are derived from HSCs.

In the current study, we found abundant efferent signals transported from the sensory nerves into the osteoclasts; furthermore, sensory hypersensitivity significantly increased osteoclastic bone resorption, and the SN directly promoted osteoclastogenesis in in vitro coculture systems. All the above results suggested that there was a direct promoting effect of sensory nerves on osteoclastogenesis. The muscle tissue consists of fast-twitch myofibers responsible for rapid bursts of contraction and slow-twitch myofibers that exert slow contractions for endurance exercises such as standing; notably, the majority of sensory efferent signals (87.28%) are present in the fast-twitch myofibers. The above results suggested that the efferent function of sensory nerves tends to be involved in the rapid feedback process. Zhu et al. found that sensory nerves and osteoclasts were increased in the subchondral bone as early as the first week after anterior cruciate ligament transection (ACLT) surgery, that is, at the early stage of osteoarthritis (OA).^44^ However, Hao et al.^8^ found that the number of osteoclasts was not altered in the mouse models of sensory denervation (TrkA _Avil-/-_ or adult iDTR _Avil_^fl-/-^ mice injected with 1 μg·kg^−1^ diphtheria toxin). Toru et al.^45^ also observed no significant changes in osteoclasts in Sema3a _synapsin-/-_ mice, even though the number of sensory innervations in the trabecular bone was significantly decreased. The difference between these reports and our results may be because sensory denervation induces compensation of other nerves, which also regulate osteoclasts. For example, patients with hereditary sensory neuropathy had no osteoclasts in severely degenerated areas of sensory nerves.^15^ However, patients with familial dysautonomia, which is characterized by autonomic and sensory dysfunction,^46^ experience osteoporosis.^47^ Indeed, neural regulation of bone is complicated. Therefore, factors that trigger sensory nerves to directly promote osteoclastogenesis require further investigation.

In the current study, our screening identified a novel neuropeptide, Cyp40, which is an efferent signal from the sensory nerve and plays a critical role in osteoclastogenesis. It was found that sensory Cyp40, when present in osteoclasts, interacted with AhR to downregulate the Ras/c-Raf/p-Erk signaling pathway through AhR’s noncanonical activity. This finding was important since AhR, which exerts both promoting and inhibitory effects on osteoclastogenesis,^48,49^ has been primarily associated with its canonical activity.^50^ Ye et al. recently reported a noncanonical, transcription-independent AhR function^40^ in other biological processes; however, this is yet to be further explored in the context of bone metabolism. We found that sensory Cyp40 downregulated AhR expression and regulated its noncanonical activity, which was independent of its canonical function. The noncanonical activity of AhR, which is involved in the regulation of Erk signaling,^40^ has been linked to the regulation of NFATc1, a transcription factor that is crucial for osteoclast-specific gene expression.^35–37,51,52^ Notably, AhR downregulation resulted in the downregulation of Ras/c-Raf/p-Erk signaling as well. Consequently, this process relieves the inhibitory effect of p-Erk on NFATc1, ultimately leading to an increase in osteoclastogenesis.

The Cyp40 protein has not been studied extensively. Previous research on Cyp40 primarily focused on its chaperone features and its role in regulating the function of hormone-like receptors, including steroid receptors,^53–59^ estrogen receptors^60,61^ and AhR,^39,62^ in participating in stress responses.^63–67^ Although our findings demonstrate that Cyp40 inhibits the Ras/c-Raf/p-Erk signaling pathway in osteoclasts by interacting with AhR, it is plausible that Cyp40 functions as a chaperone protein by binding to other factors that are critical for the genesis, differentiation, and activity of osteoclasts. These factors could include various signaling molecules, transcription factors, or cofactors that are involved in the regulation of osteoclastic functions. Cyp40 may not play a direct causal role in these processes due to its chaperone activity.

Collectively, this study provides direct evidence that sensory nerves can promote osteoclastogenesis by secreting the neuropeptide Cyp40, which involves mediation of the AhR-Ras/Raf-p-Erk-NFATc1 signaling pathway by Cyp40. These findings provide new insights into a novel mechanism of the effect of sensory nerves on osteoclasts, suggesting Cyp40 inhibition as a potential strategy to improve bone quality in osteoporosis and promote bone repair after bone injury.

## Materials and methods

### Animals and drug administration

Sprague‒Dawley (SD) rats and BALB/c mice were obtained from the Experimental Animal Center of Air Force Medical University. GFP^+^ SD rats were purchased from Xing Ming Biomedical Technology Co., Ltd. (Shanghai, China). All animal procedures were approved by the Committee for the Care and Use of Laboratory Animals of Air Force Medical University and were performed in an authorized animal care facility.

### RTX

Three escalating doses of resiniferatoxin (RTX, VIRTUE-CLARA, VTY25831), e.g., 10 μg·kg^−1^, 20 μg·kg^−1^, and 30 μg·kg^−1^, were injected subcutaneously into 4-week-old C57BL/6J mice on 3 consecutive days. The control littermates were injected with a vehicle solution on the same days.

### Cells

BMSCs, bone marrow monocytes (BMMCs), and GFP^+^ BMMCs. BMSCs,^68^ BMMCs, and GFP^+^ BMMCs^69^ were isolated from the femur bone marrow of 2-week-old WT and GFP^+^ SD rats, as published previously.

### Sensory neurons

SNs were obtained from embryonic Day 15 (E15) Sprague-Dawley rat embryos using a procedure that was published previously.^70^ The embryos were extracted from the uterine horns of the pregnant rats, and their spinal cords were carefully harvested to collect the DRGs attached to the sides of the cord. The freshly obtained DRG tissues were placed in a 100 mm dish containing high-glucose α-MEM medium and kept on ice. After sampling, the DRG tissues were gently washed three times with PBS and then transferred to a 6-well plate. A digestion solution composed of 1 mL of type IV collagenase, 1 mL of trypsin, and 4 mL of high-glucose α-MEM was added to the tissue. The tissue was finely minced using ophthalmic scissors and digested at 37 °C for 30 min. Subsequently, the digestion solution containing the digested tissue was sieved, centrifuged, resuspended, and cultured in neural medium with 10 μmol·L^−1^, 5-fluoro-2-deoxyuridine (FdUrd), and 10 μmol·L^−1^ uridine for 48 h. The culture medium was then replaced with regular neural medium (without FdUrd and uridine) for continued cultivation.

### Lentiviral and plasmid vectors

SNs were transfected with overexpression plasmids or infected with lentiviral knockdown vectors, as described previously.^71^ For overexpression of Cyp40, the SNs were transfected with the pEGFP-N1 plasmid (Fig. S4a) containing the gene that encodes Cyp40 (PPID; lv-Cyp40). The sequences of primers used to detect lv-Cyp40 were as follows: 5’-CGCAAATGGGCGGTAGGCGTG-3’ and 5’-CGTCGCCGTCCAGCTCGACCAG-3’. For the knockdown of Cyp40 and Mif, the SN was infected with lentiviral vectors (GV298, U6-MCS-Ubiquitin-Cherry-IRES-puromycin) that targeted Cyp40 (shCyp40) or Mif (shmif). The shCyp40 target sequence was CCTGCTAAAGGCTGTGATCAA, and the shmif target sequence was CCTGCACAGCATCGGCAAGAT. For AhR knockdown. AhRsiRNA (directed against rat AhR mRNA sequence [gi:6978474] from position 3 291 to 3 309): CGUUAGAUGUUCCUCUGUGTT (sense), and CACAGAGGAACAUCUAACGTT (antisense); with lentiviral vectors rLV-U6-shRNA (AhR)-CMV-mCherry-2a-Puromycin.

For signal tracing from the sensory nerve to the bone in vivo, 700 nL of VSV-EGFP (BrainVTA, v01001) was injected into the L3/L4 DRG.

### Tartrate-resistant acid phosphatase (TRAP) staining

For detection of osteoclast differentiation in vitro, BMMCs were seeded into new dishes at 5.0 × 10^4^ cells per mL. The cells were then cocultured with SNs or modified SNs wherein Cyp40 or Mif had been overexpressed or knocked down. For cells with no intervention (control group) or cells that had been treated with the saphenous nerve homogenate, recombinant Cyp40 or recombinant Mif were used. The cells were cultured in media containing M-CSF (25 ng·mL^−1^, PeproTech) for 3 days. Thereafter, a different medium containing M-CSF (25 ng·mL^−1^) and RANKL (100 ng·mL^−1^, PeproTech) was used, which was accompanied by treatments (coculture, sensory nerve homogenate, recombinant proteins) for another 3 days. On Day 6, the cells were fixed and stained for TRAP using a leukocyte acid phosphatase kit (Sigma). TRAP^+^ osteoclasts with more than three nuclei were quantified using ImageJ software.

### Osteoclast resorption activity

Briefly, BMMCs were labeled with FACS in CaP-coated plates and seeded into the coated plates in phenol red-free α-MEM containing 25 ng·mL^−1^ M-CSF for 3 days. Thereafter, a different medium containing M-CSF and 150 ng·mL^−1^ RANKL was used, followed by different treatments (coculture, sensory nerve homogenate, recombinant proteins; see grouping strategy in TRAP staining) for another 6 days. On Day 9, 100 µL of the conditioned medium from each well was transferred to a new plate to measure the resorbing activity using a bone resorption assay kit (Cosmobio). The fluorescence intensity was measured at an excitation wavelength of 485 nm and emission wavelength of 535 nm.

### Quantitative real-time polymerase chain reaction (qPCR)

Total RNA was purified from the cells using TRIzol (Invitrogen, 15596026), reverse-transcribed using Prime Script TM RT Master Mix (TaKaRa, Japan), and subjected to qPCR using Taq SYBR Green Power PCR Master Mix (Invitrogen, A25777) on a CFX96TM real-time system (Bio-Rad). GAPDH was used as the internal control. The primer sequences were as follows: Mmp9 forward, CGTCGTGATCCCCACTTACT and reverse, AACACACAGGGTTTGCCTTC; CTSK forward, CAGTCCACAAGATTCTGGGG and reverse, GGTTCCTGTTGGGCTTTCAG; and GAPDH forward, ATGTGTCCGTCGTGGATCTGA and reverse, ATGCCTGCTTCACCACCTTCTT.

### Immunofluorescence staining

The femur specimens were dehydrated in 30% sucrose and 10% gum Arabic for 3 days at 4 °C and embedded in an optimal cutting temperature compound. Then, 10 μm-thick sections were prepared. Immunofluorescence staining was performed according to standard protocols. Briefly, sections were permeabilized in 0.2% Triton X-100 (Sigma), nonspecific binding was performed by blocking in 10% donkey serum (Solarbio), and the sections were incubated with primary antibodies against rat Vwf (ab6994, Abcam, 1:200), TRAP (ab2391, Abcam, 1:200), β3-tubulin (ab18207, Abcam, 1:2 000), NF-H (ab8135, Abcam, 1:1 000), Cyp40 (12716-1-AP, Proteintech, 1:100), Mif (ab7202, Abcam, 1:250), Cfl2 (sc-166958, Santa Cruz, 1:200), Tppp3 (sc-244483, Santa Cruz, 1:200), AhR (17840-1-AP, Proteintech, 1:100), TRPV1 (sc-398417, Santa Cruz, 1:200), CTSK (sc-48353, Santa Cruz, 1:100), Osx (ab209484, Abcam, 1:1 000), and CD45 (NB100-77417SS, Novus, 1:100), or Mtsn (sc-13122, Santa Cruz, 1:100) overnight at 4 °C. Fluorescent-conjugated secondary antibodies were used to detect fluorescent signals, followed by counterstaining with Hoechst 33342 (Sigma-Aldrich, 1 000 ×). Images of the center field of view were captured for each independent sample using a confocal microscope (A1R, Nikon), and immunofluorescence staining intensity was quantified using ImageJ software.

### Western blotting

Western blots were performed according to standard protocols. The primary antibodies included rat Cyp40 (12716-1-AP, Proteintech, 1:500), Ras (ab52939, Abcam, 1:5 000), c-Raf (ab50858, Abcam, 1:1 000), Erk (ab17942, Abcam, 1:1 000), p-Erk (ab201015, Abcam, 1:1 000), Mif (ab7202, Abcam, 1:2 000), AhR (17840-1-AP, Proteintech, 1:500), Cyp1a1 (13241-1-AP, Proteintech, 1:500), EGFP (CAB4211, Invitrogen, 1:200), CD9 (ab92726, Abcam, 1:2 000), GAPDH (ab9485, Abcam, 1:2 500) and β-actin (ab8226, Abcam, 1:1 000).

### iTRAQ

The saphenous nerves from 300 BALB/c mice were randomly divided into two groups (150 mice per group). The samples in the first group were rinsed to wash away most axoplasmic proteins (Y1), and those in the other group were untreated (Y2). More abundant components in the Y2 group were considered axoplasmic proteins. Furthermore, iTRAQ screening of the saphenous nerve axoplasm was performed by the Beijing Genomics institution, as published previously.^72^ Proteins from each sample were labeled with the iTRAQ reagent (Applied Biosystems) as follows: sample Y1-119 tags, Sample Y2-121 tags. Proteins with *P* values < 0.05 and fold changes >1.2 between groups were considered differentially expressed proteins.

### Immunoelectron microscopy (IEM)

The femurs seeded with BMMCs were isolated and cocultured with SN cells transfected with plasmids containing EGFP-tagged Cyp40. M-CSF was added to the media for the first 24 h to induce BMMC attachment. M-CSF and RANKL were then added to the media to induce the differentiation of BMMCs into osteoclasts for 5 days. The osteoclast-bone composite was fixed in 4% paraformaldehyde and 0.05% glutaraldehyde for 24 h, decalcified in 10% EDTA for 4 weeks, and sectioned using a vibratome (VT1000S, Leica) to obtain 45 μm-thick sections. These sections were washed 30 times with 0.01 mol·L^−1^ PBS to remove the fixative, blocked in 5% BSA and 0.05% Triton for 3 h, washed 15 times with PBS, incubated with the primary antibody against Cyp40 (12716-1-AP, Proteintech, 1:100) or EGFP (ab6556, Abcam, 1:1 000), diluted in 1% BSA and 0.05% Triton X for 24 h at room temperature, and washed 30 times with PBS. The sections were incubated with 1.4 nm nanogold-IgG goat anti-rabbit IgG antibody (#2003-1, Nanoprobes, 1:100) diluted in 1% BSA and 0.05% Triton for 4 h at room temperature, washed 35 times with PBS, fixed in 20 mL·L^−1^ glutaraldehyde for 20 min, and then washed 30 times in PBS followed by deionized water. The sections were incubated in an HQ Silver Enhancement Kit (#2012-45, Nanoprobes) for 15 min in the dark to enhance the sensitivity. The reaction was stopped in cold deionized water. The sections were then washed 30 times in cold deionized water, washed 30 times in phosphate buffer (PB), fixed in 5 mL·L^−1^ citric acid for 1.5 h, and washed with PB. The washed sections were dehydrated in an ethanol gradient from 300 mL·L^−1^ to 1 000 mL·L^−1^ while immersed in acetone: Epon812 (1:1) for 45 min, immersed in Epon812 for 12 h, and then flat embedded and polymerized at 60 °C for 24 h. Ultrathin sections were then obtained using an ultramicrotome (EM UC6, Leica) and stained with uranyl acetate and lead citrate. Transmission electron microscope images were captured and analyzed using a JEM-1230 (JEOL) with Gatan Digital Micrograph 3.9.

### Exosomes

The medium was collected from the coculture system and centrifuged at 500 × *g* for 5 min to remove cellular components, which was followed by centrifugation at 2 000 × *g* for 10 min to remove cellular debris and another centrifugation at 10 000 × *g* for 30 min to remove large particle particles. The supernatant was filtered through a 0.22 µm filter and centrifuged at 100 000 × *g* for 70 min, and the (nonexosomal) supernatant was collected (MS). The exosomes in the pellet were resuspended in PBS and transferred to a new centrifuge tube.

### Coimmunoprecipitation

The cells were lysed in IP lysis buffer (Thermo Scientific, #87787) for 1 h and incubated with PureProteome Protein A or Protein G Magnetic Beads (Millipore, #LSKMAGA02) as well as antibodies against AhR (17840-1-AP, Proteintech, 1:50) and GFP (ab6556, Abcam, 1:100) at 4 °C overnight. The immunoprecipitates were subjected to immunoblotting.

### Three-point bending tests

For measurement of the bone strength of the femurs, the BOSE Electroforce (3220) was used to perform a three-point bending test. The femur specimen was placed steadily on the bending jig such that the short axis of the femur was consistent with the direction of the force. The span of the two fulcrums was 8 mm, the preload was 0.5 N, and the loading speed was 0.02 mm·s^−1^. Furthermore, the route was 2 mm, and the test was terminated after the specimen was destroyed. The biomechanical measurement data were collected from the load‒deformation curves. The maximum load (N) was recorded.

### Biochemical parameters

Detection of biochemical parameters in the serum was performed using an automatic biochemical analyzer (MS-480) and matching kits. The parameters that were investigated included total inorganic phosphate (P, 201SJTZ306), cholesterol (TCH, 201SJTZ202), triglycerides (TG, 201SJTZ201), glucose (Gluhk, 201SJTZ108), creatine kinase (CK, 201SJTZ006), urea nitrogen (Urea, 201SJTZ106), creatinine (CR, 201SJTZ105), and uric acid (UA, 201SJTZ107).

### Supplementary information

supplement excel1 iTRAQ -secretory proteins in sensory nerve Y1-VS-Y2_Down

supplement excel2 iTRAQ -Y1-VS-Y2_Up

Supplement Materials
