# Supplementary material for: Sensory nerves directly promote osteoclastogenesis by secreting peptidyl-prolyl cis-trans isomerase D (Cyp40)
Source: Bone Res. 2023 Dec 14;11:64. doi: 10.1038/s41413-023-00300-w (PMC10721806; doi:10.1038/s41413-023-00300-w)
Supplement: Supplementary file 3 — Supplement Materials [file 41413_2023_300_MOESM3_ESM.docx]

**Supplement Materials**


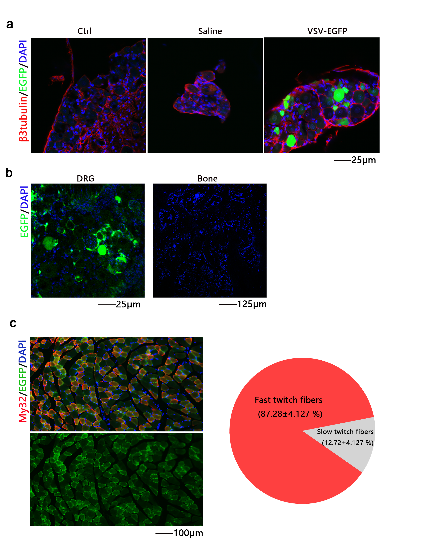


**Fig S1. Signals from sensory nerve into muscle**

(a) Representative confocal image target β3tubulin in the dorsal root ganglion (DRG). L3/L4 DRG were infected with VSV-EGFP vectors; Control littermates were injected with saline (saline) or nothing just the sham operations (ctrl) on the same days; (b) The distribution of EGFP signals in the bone and DRG after VSV-EGFP vector injection for 24 hours; (c) Representative confocal image target My32 in the muscle. N.S. means not significant, versus controls, Student’s t test. The results are expressed as the mean ± s.d.

**
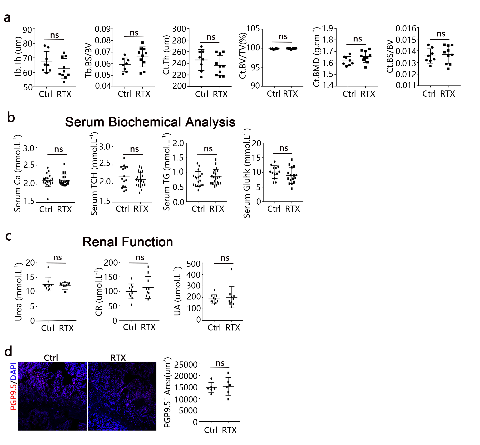
**

**Fig S2. Quantitative analyses of tibia and biochemical parameters in the RTX treatment mice (versus control mice)**

(a) Quantitative analyses of trabecular bone thickness (Tb.Th) , cortical bone thickness (Ct.Th), cortical bone volume/tissue volume (Ct. BV/TV), cortical bone mineral density (Ct. BMD), and cortical bone surface per bone volume (Ct.BS/BV)of tibia by micro-computed tomography (μCT); (b) Serum biochemical analyses ; (c) The detection of serum biomarkers for kidney function. N.S. means not significant, versus controls, Student’s t test. The results are expressed as the mean ± s.d; (d) Representative confocal images and quantitative analysis of PGP9.5.in bone.


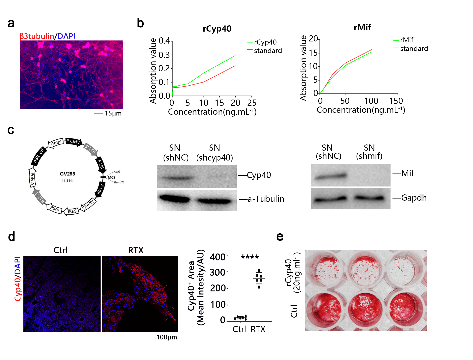


**Fig S3. Both Mif and Cyp40 in DRG are down-regulated**

(a) Representative confocal image target β3tubulin of sensory neurons; (b) ELISA tests were performed to assess the activity of recombinant Cyp40 and Mif (n=4); (c) Schematic of the GV298 vector. Western blots to confirm the knockdown efficiency (shCyp40, shMif) of Mif and Cyp40 in sensory neurons; (d) The basal expression of Cyp40 in sensory neurons and bone cells; (e)Representative images of alizarin red staining (n=3). *P < 0.05, **P < 0.01, ***P < 0.001, and N.S. means not significant, versus controls, Student’s t test. The results are expressed as the mean ± s.d.


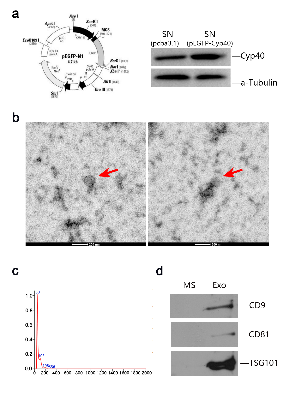


**Fig S4. Characterization of exosomes**

(a) CYP40 was upregulated and labeled with an EGFP tag. Schematic of the pEGFP-N1 vector. Western blots to confirm overexpression of Cyp40 (pEGFP-N1/Cyp40) in sensory neurons ;(b) Representative TEM images of exosomes (red arrow); (c) Particle size distribution of purified vesicles; (d) Western blots target CD9, CD81, TSG101 in exosomes (Exo) and medium supernatants (MS).
